# Effects and related mechanisms of serotonin on malignant biological behavior of hepatocellular carcinoma via regulation of Yap

**DOI:** 10.18632/oncotarget.17658

**Published:** 2017-05-07

**Authors:** Sushun Liu, Runchen Miao, Mimi Zhai, Qing Pang, Yan Deng, Sinan Liu, Kai Qu, Chang Liu, Jingyao Zhang

**Affiliations:** ^1^ Department of Hepatobiliary Surgery, The First Affiliated Hospital of Xi’an Jiaotong University, Xi’an, China; ^2^ Department of General Surgery, The Second Xiangya Hospital, Central South University, Changsha, China; ^3^ Department of SICU, The First Affiliated Hospital of Xi’an Jiaotong University, Xi’an, China

**Keywords:** 5-HT, Yap, 5-HT_2B_R, ERK, hepatocellular carcinoma

## Abstract

5-hydroxytryptamine (5-HT, serotonin) and Yes-associated protein (Yap), which act as a mitogen and an oncogene, respectively, play an important role in tumors. Here, we investigated whether 5-HT could affect the hepatocarcinogenic process via promoting the activation and expression of Yap, as well as the possible underlying molecular mechanisms. We found that 5-HT promoted hepatoma cell proliferation, invasion and metastasis via regulating Yap expression *in vitro* and *in vivo*, and Yap knockdown had opposite effects. Furthermore, 5-HT activated 5-HT_2B_R to promote Yap expression via upregulating the pERK level. Inhibitors of 5-HT_2B_R and ERK attenuated the overexpression of Yap and promotional effects of 5-HT *in vitro* and *in vivo*. As a result, 5-HT affected the malignant biological behavior of hepatoma cells via the 5-HT-5-HT_2B_R-pERK-Yap axis. Therefore, 5-HT and Yap may be prognostic predictors and potential therapeutic targets for HCC patients in the future.

## INTRODUCTION

In China, hepatocellular carcinoma (HCC) has a high incidence and mortality that are still increasing, causing a huge impact on national health. Recent studies of 5-HT have indicated the association between 5-HT and cancer [1–7]. Increasing evidence has shown that 5-HT has serum-like effects on certain types of hepatoma cells to promote proliferation, invasion and metastasis [5, 7]. Moreover, animal experiments also confirm the promoting effects of serotonin in xenograft models [5, 7].

Landmark studies have implicated that the activation of the Hippo-Yap signaling pathway influences liver cell fate [8]. Engagement of the Hippo signaling pathway results in inactivation and phosphorylation of Yap [9]. By contrast, Yap localizes to the nucleus and exerts its transcriptional activity mainly by interacting with the TEAD [10]. As an oncogene, Yap is associated with many types of tumors [11–17]. Although the Hippo-Yap signaling pathway plays a critical role in organ size regulation and tumorigenesis, rare study is available on the regulation of Yap via the Hippo-independent signaling pathway.

In this study, we investigated whether 5-HT could affect the hepatocarcinogenic process via promoting activation and expression of Yap, as well as the possible underlying molecular mechanisms. We found that 5-HT activated the 5-HT_2B_ receptor to promote expression of Yap via upregulation of the pERK level. Our findings suggest that the 5-HT-Yap pathway acts as a tumor activator in HCC and it might be a potential therapeutic target for HCC patients.

## RESULTS

### 5-HT promotes the malignant biological behavior of hepatoma cells via regulating Yap expression

**Figure 1:**
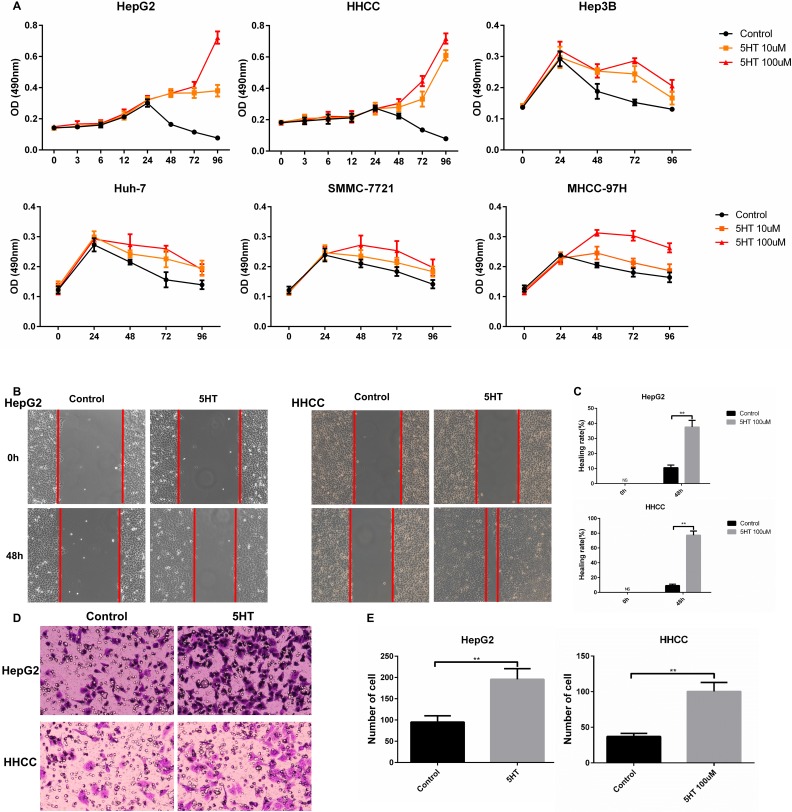
5-HT promotes the proliferation, invasion and metastasis of hepatoma cells (**A**) 5-HT significantly promoted HepG2 and HHCC cell proliferation in SFM as evaluated by the MMT assay at the indicated times. (**B**–**C**) 5-HT induced faster wound closure in HepG2 and HHCC cells (B), and the results were quantified using the healing rate (C). (**D**–**E**) 5-HT induced more cell penetration at 24 h in HepG2 and HHCC cells (D), and the penetrated cells were counted (E). *P < 0.05, **P < 0.01.

Previous studies have shown that 5-HT exerted serum-like effects on certain types of hepatoma cells [5, 7]. In our study, 5-HT elevated the viabilities of hepatoma cells, especially serum-deprived HepG2 and HHCC cells. Additionally, medium containing 100 μM serotonin exhibited the strongest promotion effect (Figure 1A). Additionally, faster wound closure (Figure 1B–1C) and more cell penetration (Figure 1D–1E) were observed in HepG2 and HHCC cells cultured with serotonin.

**Figure 2:**
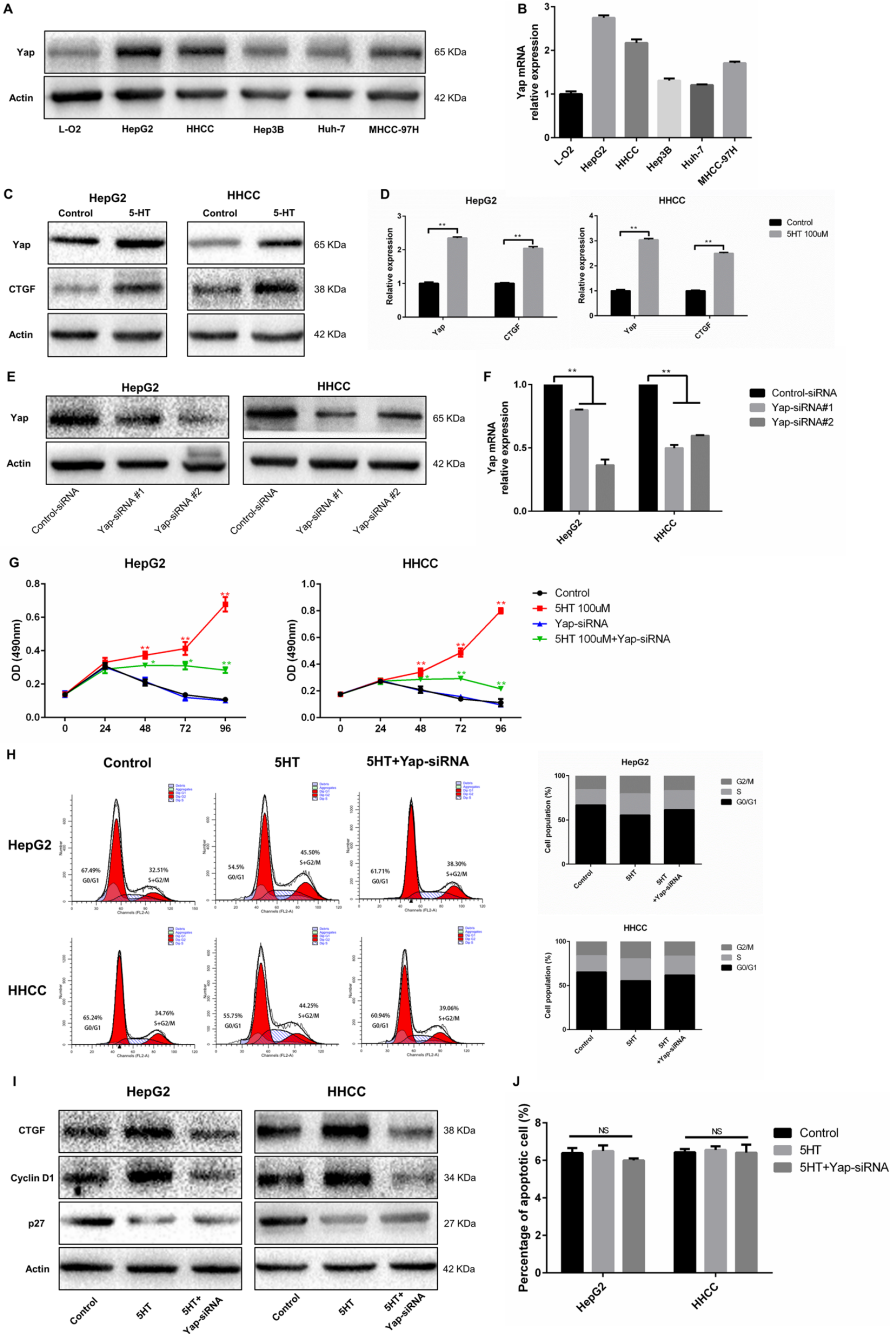
Yap, which is regulated by 5-HT, promotes proliferation (**A**–**B**) Yap expression in hepatoma cells was higher than those in normal hepatocytes in the presence of 5-HT at the protein (A) and mRNA levels (B) and was especially higher in HepG2 and HHCC cells among the hepatoma cells. (**C**–**D**) 5-HT promoted Yap expression in HepG2 and HHCC at the protein (C) and mRNA levels (D). (**E**–**F**) Yap expression was significantly downregulated using Yap-siRNA at the protein (E) and mRNA levels (F) in HepG2 and HHCC cells. (**G**) Inhibition of Yap inhibited the proliferation promotion effect induced by 5-HT. (H-I) Yap inhibition reduced the S+G2/M phase cell ratio in HepG2 and HHCC cells (**H**), and the marker proteins of the cell cycle were also detected (**I**). (**J**) 5-HT administration and Yap inhibition did not affect cell apoptosis. Red asterisk: control group vs 5-HT group; Green asterisk: 5-HT group vs 5-HT+Yap-siRNA group; **P < 0.01.

In order to explored the promoting effects of 5-HT on hepatoma cell, we investigated the promotion effects of 5-HT on Yap expression. Among the cells, L-O2 showed the lowest Yap expression among the selected cells (Figure 2A–2B). Additionally, the HepG2 and HHCC showed the highest Yap expression (Figure 2A–2B). To further validate the promotion effects of 5-HT on Yap expression, the expression of Yap in HepG2 and HHCC cells in the absence or presence of 100 μM serotonin was assessed. Additionally, Yap and Connective tissue growth factor (CTGF, downstream target of Yap) expression levels were higher with the administration of 5-HT than those in the control group (Figure 2C–2D).

**Figure 3:**
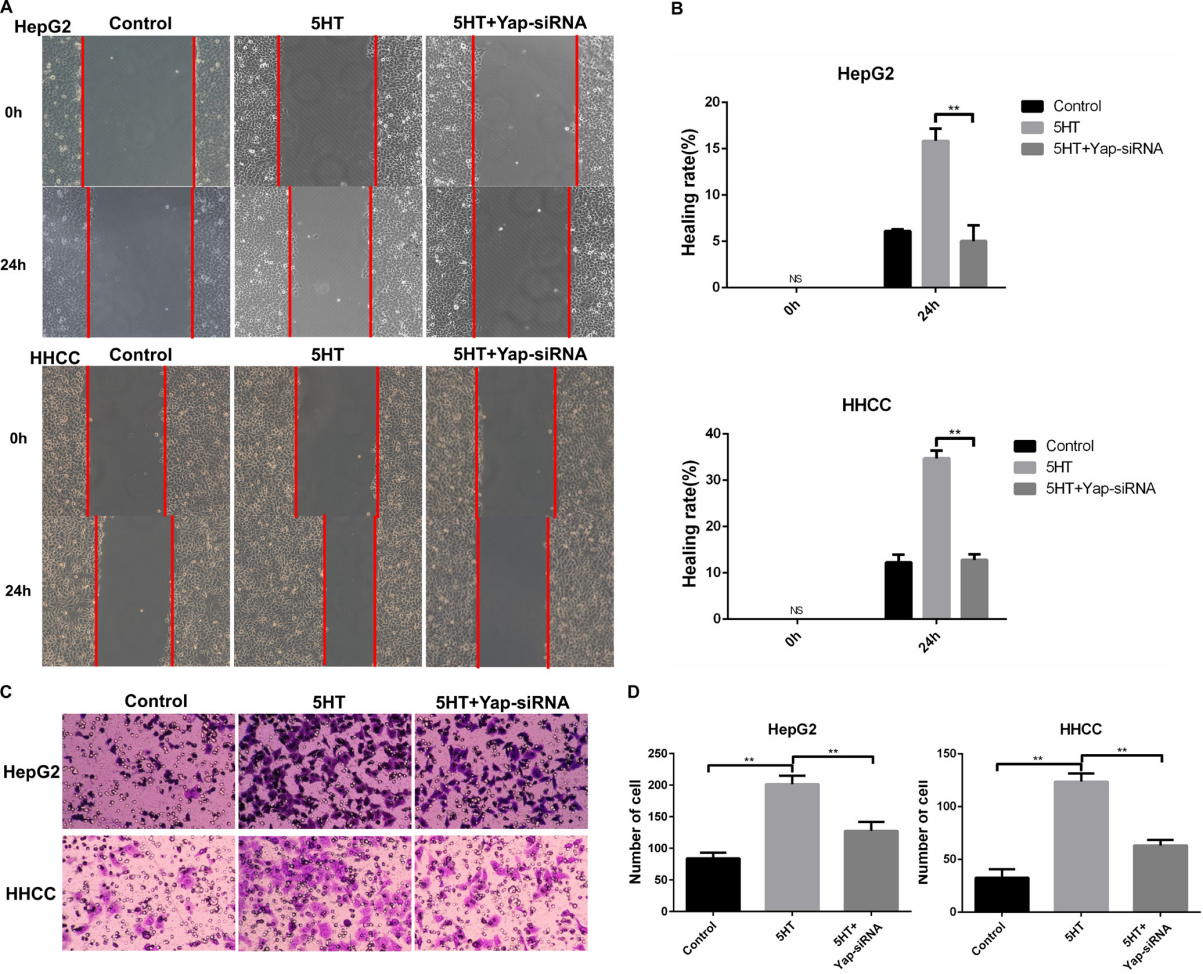
Yap promoted invasion and metastasis of HepG2 and HHCC cells (**A**–**B**) Inhibition of Yap induced slower wound closure in HepG2 and HHCC cells (A), and the healing rate was used for quantification (B). (**C**–**D**) Inhibition of Yap induced less cell penetration (C), and the penetrated cells were counted for quantification (D). ns: no statistical significance, **P < 0.01.

Next, we ascertained whether Yap acted as a downstream target of 5-HT in hepatoma cell. After transfection with Yap-siRNA, HepG2 and HHCC cells were cultured in SFM (serum-free medium) containing 5-HT. First, we explored the efficacy of transfection of the two types of Yap-siRNA (Figure 2E–2F). Next, the cell proliferation and cell cycle of HepG2 and HHCC cells transfected with Yap-siRNA were analyzed. As a result, Yap-siRNA significantly reduced HepG2 and HHCC cell proliferation in the presence of 5-HT (Figure 2G). Moreover, the S+G2/M phase cell ratio was increased in the presence of 5-HT and was significantly reduced in Yap-siRNA-transfected hepatoma cells (Figure 2H–2I). However, cell apoptosis was not affected by 5-HT or Yap-siRNA transfection (Figure 2J). To determine the potential metastatic promotion effect of Yap affected by 5-HT, we investigated cell motility and invasion abilities. Compared with the HepG2 and HHCC cells in the presence of 5-HT, slower wound closure (Figure 3A–3B) and less cell penetration (Figure 3C–3D) were observed in Yap-siRNA-transfected cells.

### 5-HT_2B_ receptor activated by 5-HT upregulates Yap expression

**Figure 4:**
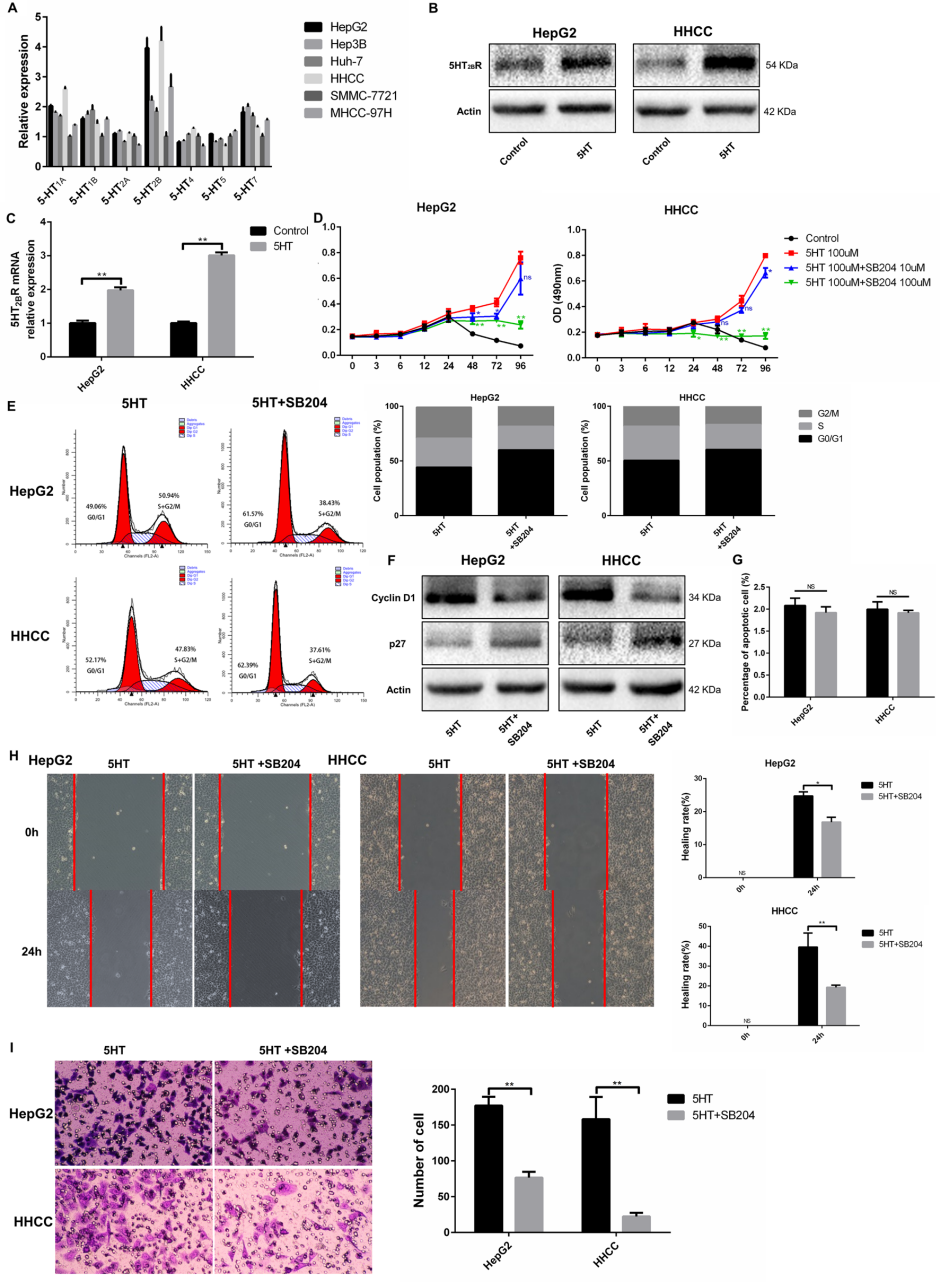
5-HT_2B_R promoted the proliferation, invasion and metastasis of HepG2 and HHCC cells (**A**) The expression of 5-HT receptors in different types of hepatoma cells. The 5-HT receptors expression of SMMC-7721 were considered as 1. (**B**–**C**) 5-HT promoted the expression of 5-HT_2B_R in HepG2 and HHCC at the protein (B) and mRNA levels (C). (**D**) Inhibition of 5-HT_2B_R inhibited the promotion effect on proliferation induced by 5-HT. (**E**–**F**) Inhibition of 5-HT_2B_R reduced the S+G2/M phase cell ratio in HepG2 and HHCC cells (E), and cell cycle marker proteins were also detected (F). (**G**) 5-HT administration and 5-HT_2B_R inhibition did not affect cell apoptosis. (**H**) Inhibition of 5-HT_2B_R induced slower wound closure in HepG2 and HHCC cells, quantified by the healing rate. (**I**) Inhibition of 5-HT_2B_R induced less cell penetration, quantified by the number of penetrated cells. Blue asterisk: 5-HT group vs 5-HT+SB204 10 μM; Green asterisk: 5-HT group vs 5-HT+SB204 100 μM; ns: no statistical significance, *P < 0.05, **P < 0.01.

Accumulated studies have shown that the 5-HT_2B_ receptor plays an important role in HCC [5–7]. We investigated the expression of 5-HT receptors in hepatoma cells via qRT-PCR. 5-HT_2B_R expression was the highest among all the receptors (Figure 4A). Next, we investigated whether 5-HT could affect 5-HT_2B_R expression and found that 5-HT_2B_R expression was significantly upregulated by 5-HT administration (Figure 4B–4C).

To investigate whether Yap expression was regulated by activated 5-HT_2B_R, SB204741, an inhibitor of 5-HT_2B_R, was employed. As a result, SB204741 significantly reduced the cell viability, especially at the concentration of 100 μM SB204741 (Figure 4D). Moreover, HepG2 and HHCC cells treated with SB204741 lowered the S+G2/M phase cell ratio compared with the cells only in the presence of 5-HT (Figure 4E–4F). Interestingly, SB204741 did not affect the apoptosis of HepG2 and HHCC cells, a finding that was similar to Yap-siRNA effects (Figure 4G).

As SB204741 led to inhibition of proliferation, we investigated whether SB204741 was involved in invasion and metastasis. As a result, SB204741 not only induced slower wound closure but also less cell penetration at 24 h (Figure 4H–4I).

**Figure 5:**
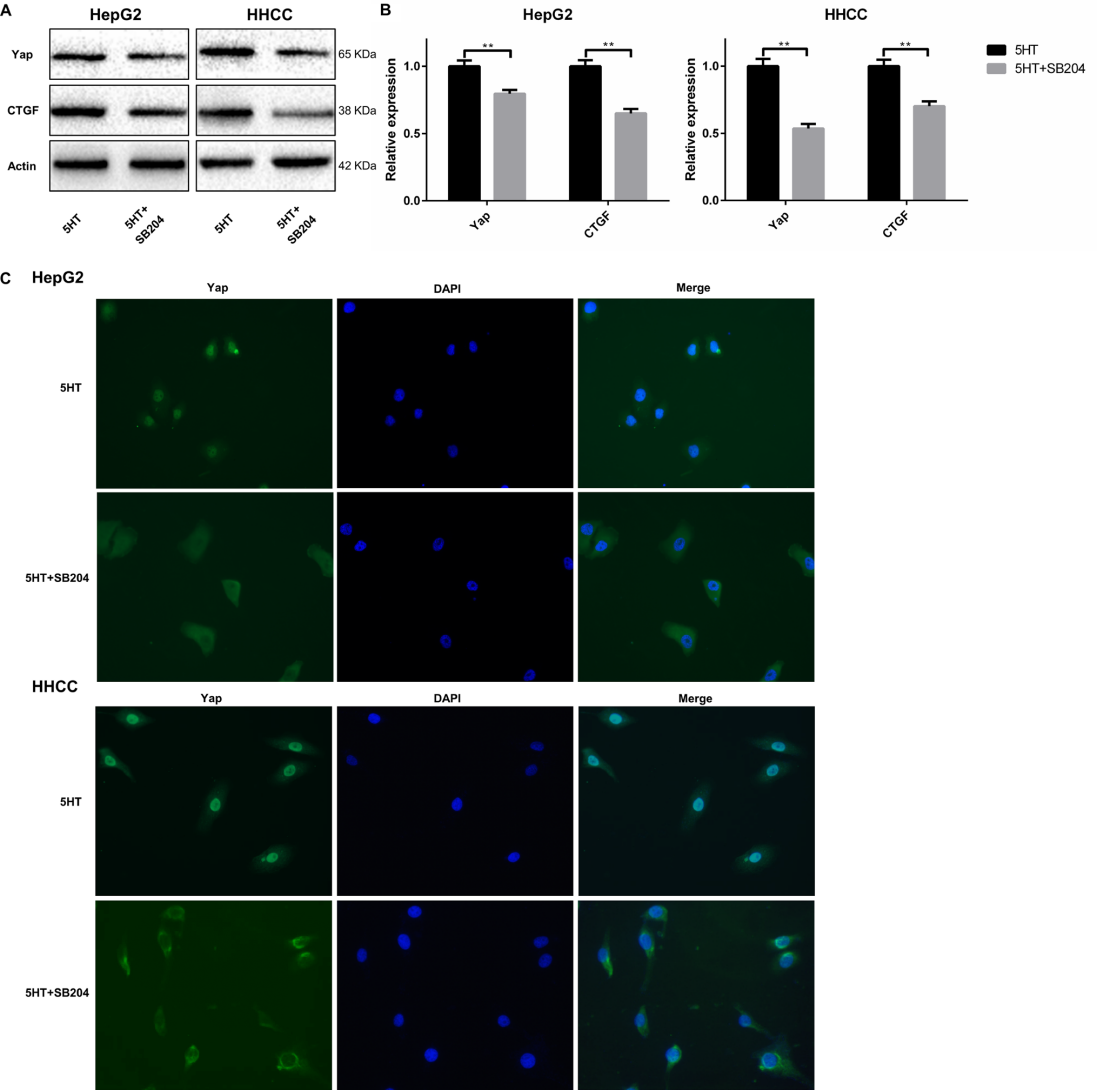
5-HT_2B_R promoted the activation and expression of Yap (**A, B**) The expression levels of Yap and CTGF (a Yap target) were downregulated by SB204741 at the protein (A) and mRNA levels (B). (**C**) 5-HT induced the entry of Yap into the nucleus, and the inhibition of 5-HT_2B_R led to the retention of Yap in the cytoplasm and inactivation of Yap. **P < 0.01.

Finally, we investigated whether activated 5-HT_2B_R could promote Yap expression. As a result, the expression of Yap and CTGF at the mRNA and protein levels were both inhibited by SB204741 (Figure 5A–5B). Additionally, immunofluorescence was used to detect the subcellular localization of Yap after culture in SFM containing 5-HT with or without SB204741. We found that Yap was predominantly expressed in nuclei in the presence of 5-HT, whereas it was mainly located in the cytoplasm after administration of SB204741 (Figure 5C).

### pERK, which is upregulated by activated 5-HT_2B_R, promotes Yap activation

**Figure 6:**
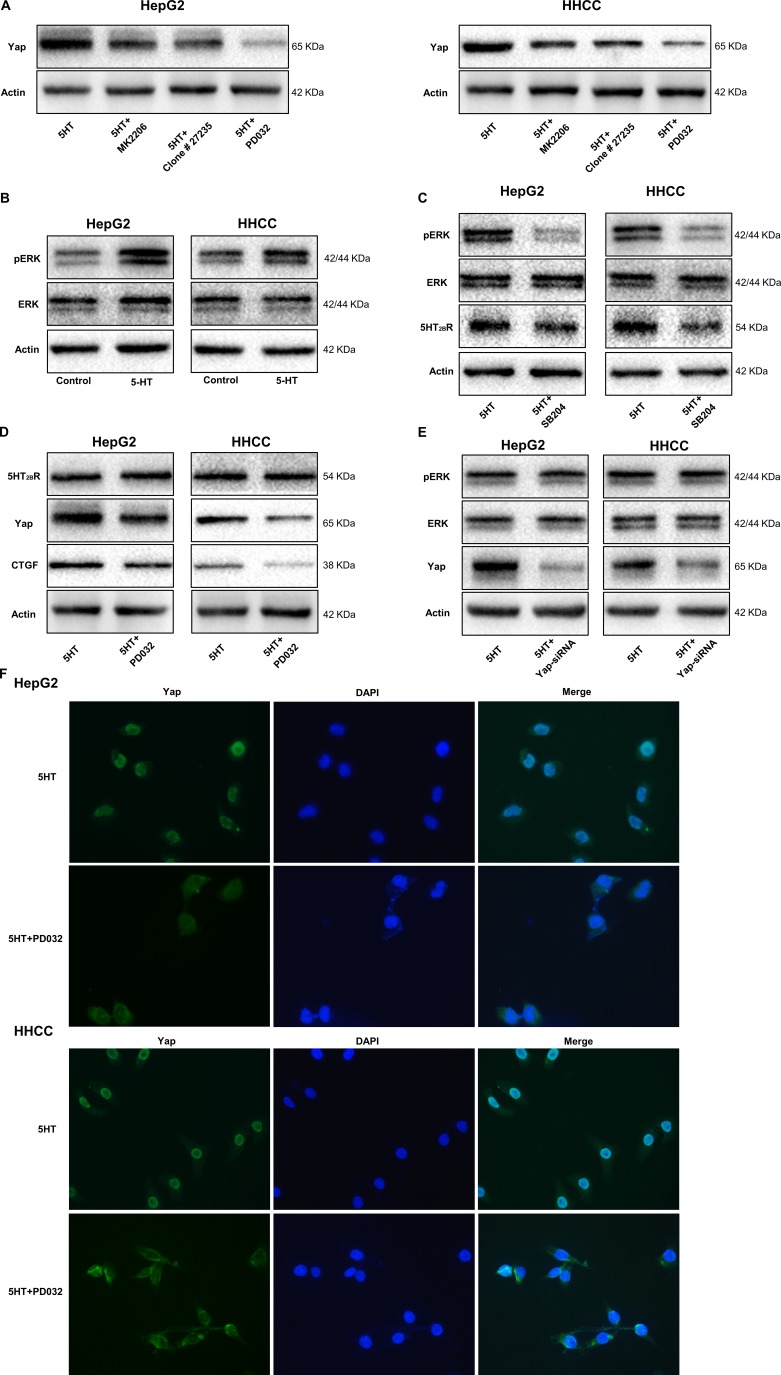
pERK upregulated by activated 5-HT_2B_R promotes Yap activation and expression (**A**) PD0325901, an inhibitor of ERK, showed the greatest inhibition of Yap expression induced by 5-HT among the inhibitors. (**B**) pERK expression was promoted by 5-HT in HepG2 and HHCC cells. (**C**) The expression levels of pERK and 5-HT_2B_R were inhibited by SB204741 in HepG2 and HHCC cells in the presence of 5-HT. (**D**) The Yap and CTGF expression levels were inhibited by PD0325901; however, the expression of 5-HT_2B_R was not affected. (**E**) Only Yap expression was inhibited by Yap-siRNA in the HepG2 and HHCC cells. (**F**) 5-HT induced the entry of Yap into the nucleus, and the inhibition of ERK led to the retention of Yap in the cytoplasm and inactivation of Yap.

To investigate the factors involved in the 5-HT-Yap pathway, inhibitors of AKT, ERK and neutralizing antibodies to TGF-β were used. As shown in Figure 6A, the expression of Yap were all inhibited by the inhibitors, and PD0325901(an inhibitor of ERK) had the best inhibitory effect (Figure 6A). Moreover, the pERK level was upregulated by 5-HT, indicating that pERK might be a target of 5-HT (Figure 6B).

To definite the relationship among 5-HT_2B_R, pERK and Yap, SB204741(an inhibitor of 5-HT_2B_R), PD0325901 and Yap-siRNA were used. Firstly, the pERK and ERK expression levels were measured in cells cultured in medium containing 5-HT with or without SB204741. As a result, the expression levels of 5-HT_2B_R and pERK were significantly inhibited by SB204741 (Figure 6C). However, PD0325901 only inhibited the expression of pERK, Yap and CTGF (Figure 6D). Additionally, the inhibition of Yap did not affect pERK and 5-HT_2B_R expression (Figure 6E). Moreover, immunofluorescence showed the similar results and we found that Yap was predominantly expressed in nuclei in the presence of 5-HT, whereas it was mainly located in the cytoplasm after administration of the inhibition of ERK (Figure 6F). Thus, we hypothesized that 5-HT affected the malignant biological behavior of hepatoma cells via the 5-HT-5-HT_2B_R-pERK-Yap axis.

### The 5-HT-Yap axis promotes the malignant biological behavior of hepatoma cells by activating pERK and Yap *in vivo*

**Figure 7:**
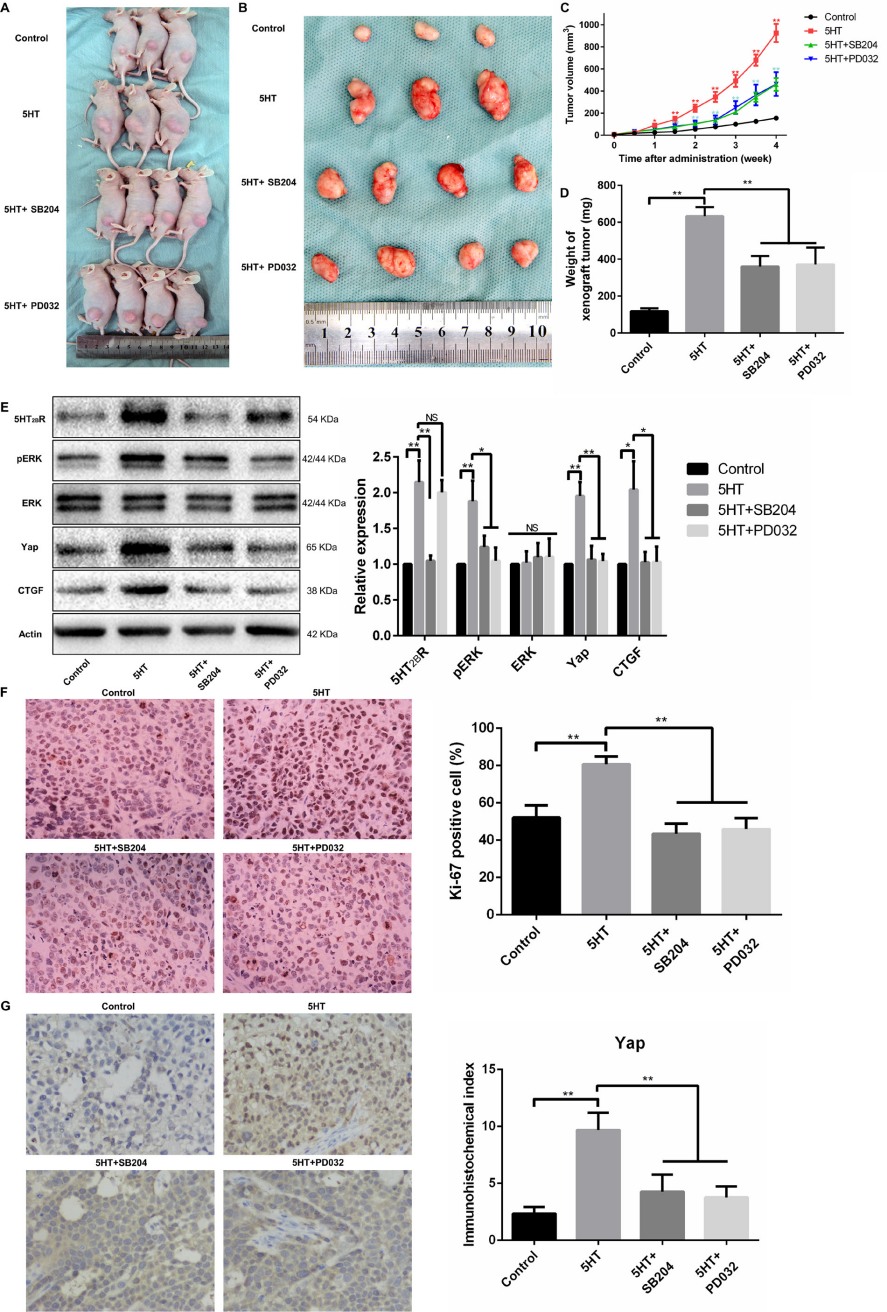
The 5-HT-Yap axis promoted the malignant biological behavior of hepatoma cells by activating Yap *in vivo* (**A, B**) Representative figures of xenograft tumors from HepG2-injected mice treated with SB204741 or PD0325901 in the presence of 5-HT. (**C, D**) The tumor volume (C) and xenograft tumor weight (D) demonstrated that both SB204741 and PD0325901 significantly inhibited tumor growth induced by 5-HT *in vivo*. (**E**) The expression levels of Yap and CTGF were significantly inhibited by SB204741 and PD0325901. Additionally, the expression levels of 5-HT_2B_R, pERK and Yap were inhibited by SB204741; however, only the pERK and Yap expression levels were inhibited by PD0325901. (**F**) The percentage of Ki-67-positive cells in the groups treated with SB204741 or PD0325901 was less than that in the 5-HT group. (**G**) 5-HT promoted the nuclear expression of Yap, and SB204741 or PD0325901 showed inhibition effects on the effects induced by 5-HT.

Because the *in vitro* study has shown that 5-HT promotes proliferation, invasion, and metastasis via the 5-HT-5-HT_2B_R-pERK-Yap axis, we further investigated the function of the 5-HT-Yap axis *in vivo*. As a result, the tumor volume and weight of mice with 5-HT administration was larger than the control group and became smaller after treatment with SB204741 or PD0325901 (Figure 7A–7D). Additionally, SB204741 inhibited the expression levels of 5-HT_2B_R, pERK, Yap and CTGF, and the expression levels of pERK, Yap and CTGF were also inhibited by PD0325901 except for the 5-HT_2B_R expression level (Figure 7E). Next, immunohistochemistry of Ki67 and Yap was conducted to investigate the proliferation ability and activation of Yap *in vivo*. As predicted, 5-HT promoted cell proliferation *in vivo*, and the proliferation promotion effects induced by 5-HT were attenuated by SB204741 and PD0325901 (Figure 7F). Importantly, the nuclear expression of Yap induced by 5-HT was also inhibited after using SB204741 or PD0325901 (Figure 7G).

## DISCUSSION

As a neurotransmitter in the central nervous system, peripheral serotonin (95%) was shown to regulate the digestive system, respiratory system, cardiovascular system and immune system, as well as promoting the proliferation of different cell types [5, 7]. However, the mechanisms by which serotonin acted as a mitogen were still confusing. Moreover, whether serotonin acted as a mitogen through a receptor-dependent or receptor-independent pathway was worth exploring. In this study, we demonstrated that serotonin upregulated the pERK level via activating 5-HT_2B_R and revealed that Yap was a key downstream effector in the 5-HT-5-HT_2B_R-pERK-Yap axis.

Yap was a co-activator of the Hippo signaling pathway. Overexpression of Yap could aberrantly activate different target genes, including CTGF, AREG, and Gli, to promote proliferation, migration and survival [18, 19]. Additionally, Yap was related to diverse tumors [11, 20, 21]. However, the relationship between serotonin and Yap was poorly understood. Interestingly, we found serotonin promoted the Yap expression and cancer process and the results were further confirmed by animal experiments. Yap expression was, no doubt, higher in hepatoma cells in the presence of serotonin. Additionally, Yap was required for proliferation, invasion and metastasis of hepatoma cells. Our data suggested that inhibition of Yap via Yap-siRNA induced inhibition of proliferation, G0/G1 phase cell arrest, slower wound closure and less cell penetration. However, cell apoptosis was not affected by Yap-siRNA or SB204741, possibly because serotonin mainly activated CTGF, which was only associated with proliferation. Additionally, Yap affected cell apoptosis by interacting with p73; however, the expression of p73 was not altered by using Yap-siRNA or SB204741. In agreement with our findings, Perra *et al*. reported that Yap was accumulated in the foci of preneoplastic hepatocytes after DENA treatment, and Yap accumulation was paralleled by its target gene upregulation [20]. Additionally, a study conducted by Zhou *et al*. demonstrated that approximately 30% of HCC patients showed lower Yap phosphorylation [22]. Surprisingly, Yap activation was also regulated by the Hepatitis B virus X protein via CREB [23].

Accumulated studies have indicated that serotonin worked by binding to serotonin receptors. A study conducted by Soll *et al*. indicated that serotonin promoted hepatocyte proliferation via upregulating the expression of 5-HT_2B_R [5]. Similarly, 5-HT_2B_R was also overexpressed in liver regeneration models [24]. Additionally, Soll *et al*. found that the expression levels of 5-HT_1B_R and 5-HT_2B_R in HCC patients were 32% and 35%, respectively, and were associated with an increased proliferation index [6]. Moreover, a study conducted by Liang *et al*. demonstrated that serotonin downregulated FoxO3a to promote proliferation via activating 5-HT_2B_R [7]. In addition, numerous studies obtained similar results [5, 6, 25]. In this study, we proved that the 5-HT_2B_R activated by serotonin had the ability to promote proliferation, invasion and metastasis. Additionally, inhibition of 5-HT_2B_R by SB204741 significantly inhibited the effects of serotonin. Moreover, our study demonstrated that Yap was modulated by activated 5-HT_2B_R. Inhibition of 5-HT_2B_R using SB204741 could downregulate the expression of Yap, leading to the retention of Yap in the cytoplasm. As an upstream factor of Yap, the pERK level was also regulated by activated 5-HT_2B_R. SB204741 downregulated the expression of pERK and Yap; however, the expression of 5-HT_2B_R was not affected by PD0325901.

In addition, we observed that Yap expression was inhibited by ERK inhibition. As shown in our study, the inhibitor of ERK inactivated Yap and led to retention of Yap in the cytoplasm. However, inhibition of Yap did not affect the expression of 5-HT_2B_R and pERK. These results were similar to previous studies reporting that ERK inhibitors or ERK-siRNA downregulated the expression of Yap and AKT [26]. A study conducted by You *et al*. also demonstrated that inhibition of ERK1/2 in NSCLS cells downregulated Yap expression [27]. Also, the pERK level was considered to be associated with HCC. A study conducted by Schmitz *et al*. demonstrated that the expression of pERK and pAKT were associated with prognosis [28]. Another study conducted by Tsuboi *et al*. indicated that the expression of ERK1/2 was higher in HHC patients, and ERK1 expression was mainly expressed in the nuclei of HCC cells [29]. Furthermore, the relationship between serotonin receptors and the pERK level was also investigated. Soll *et al*. revealed that serotonin promoted the pERK level in a time-dependent manner, and inhibitors of 5-HT_1B_R or 5-HT_2B_R could significantly reduce the pERK level [6]. Collectively, our study strongly supported the notion that 5-HT affected the proliferation, invasion and metastasis of hepatoma cells via regulation of the 5-HT-5-HT_2B_R-pERK-Yap axis. Additionally, the axis of our study was confirmed by detecting the Yap expression and Ki-67 expression in subcutaneous xenograft tumor models administered SB204741 or PD0325901.

**Figure 8:**
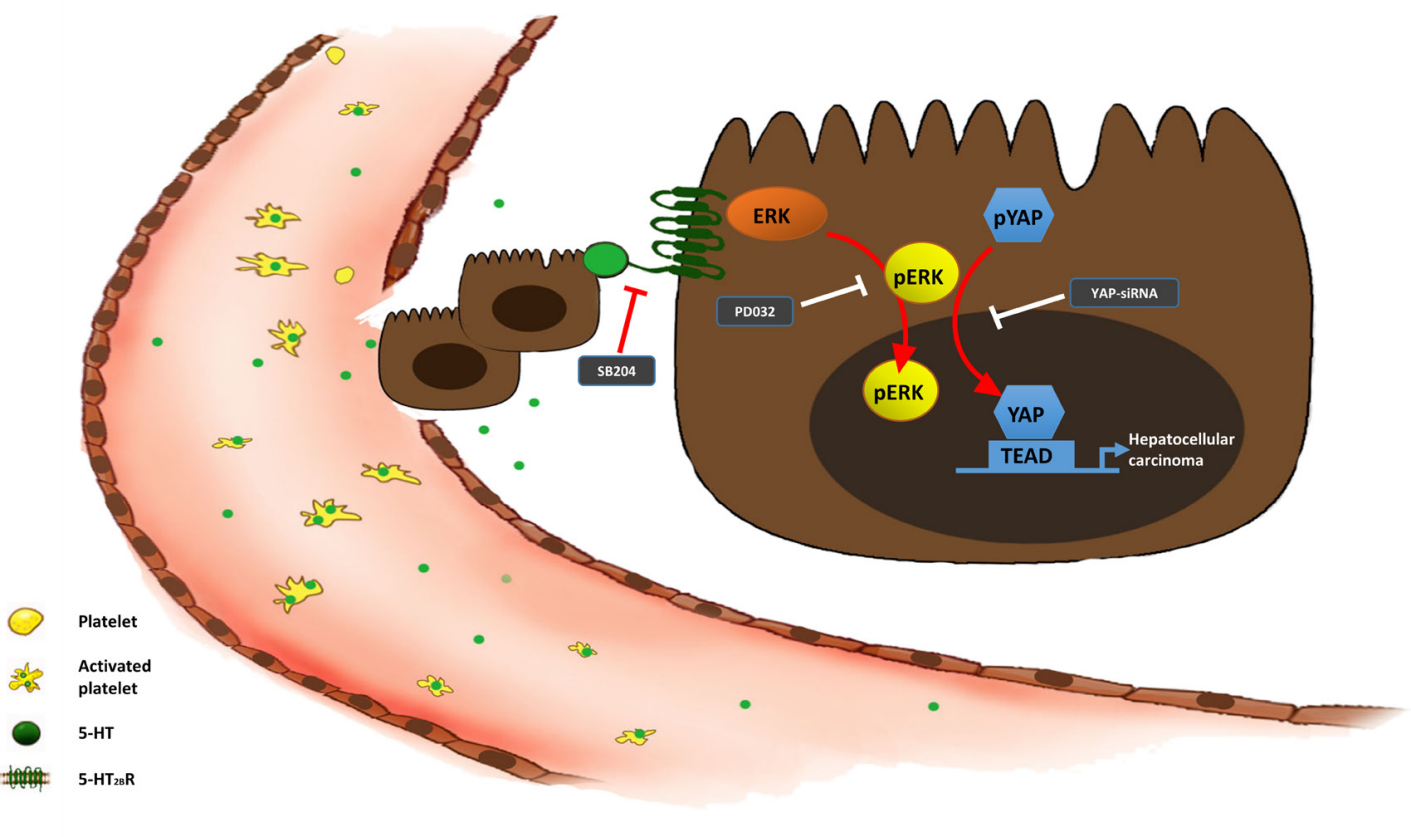
The 5-HT-Yap axis in hepatoma cells

In conclusion, the data from our study demonstrated serotonin played an essential role in proliferation, invasion and metastasis through activating 5-HT_2B_R to promote Yap expression by upregulating the pERK level (Figure 8). Our findings strongly suggested that 5-HT and Yap, which acted as a promoter of HCC, might be prognostic predictors for HCC patients. Additionally, Yap was likely to be a potential therapeutic target for HCC patients in the future.

## MATERIALS AND METHODS

### Cell lines and cell culture

The human immortalized normal hepatic cell line (L-O2) and HCC cell line (HepG2, Hep3B, Huh-7, HHCC, SMMC-7721 and MHCC-97H) were obtained from the cell bank of Chinese Academy of Sciences in Shanghai. The cells were cultured in Dulbecco's Modified Eagle's Medium (DMEM; Hyclone, South Logan, UT, USA) containing 10% fetal bovine serum (Gibco, Grand Island, NY, USA) 1% penicillin/ streptomycin at 37°C with 5% CO_2_ in incubator. During the administration, the cells were cultured only in DMEM without FBS. The 5-HT_2B_ receptor inhibitor SB204741 (Santa Cruz Biotechnology, Santa Cruz, CA, USA) and ERK inhibitor PD0325901 (Selleck Chemicals, Houston, TX, USA) were used to treat cells at concentration of 100 mM and 1 mM respectively according to the manufacturer's instructions.

The HCC cells were harvested, re-plated and cultured in incubator overnight to allow adhesion of cells. Before administration, the medium of cells was replaced by DMEM without FBS for synchronization. The groups of the study included control group (serum free medium, SFM), 5-HT group (SFM+5HT), 5-HT+SB204 group (SFM+5HT+SB204741), 5-HT+PD032 group (SFM+5HT+PD0325901) and 5-HT+Yap-siRNA group (SFM+5HT+Yap-siRNA). During the administration, the cells were pretreated in the medium containing SB204741 or PD0325901 for 30min before addition of 5-HT

### Small interfering RNA (siRNA) transfection

The human Yap-siRNA and control siRNA were synthesized by GenePharma (Shanghai, China). The siRNA sequences were shown in Supplementary Table 1.

The cells were transfected with a mixture of siRNA using Lipofectamine 2000 (Invitrogen, Carlsbad, CA, USA) according to the manufacturer's instructions. After cultured for 8h, the medium was replaced. And the knockdown efficiency of Yap was detected by qRT-PCR and Western blot assays after replacement of medium for 24 h.

### RNA isolation and quantitative real-time PCR (qRT-PCR)

Total RNA from cells and tissue samples was extracted using TRIzol reagent (Invitrogen). qRT-PCR was performed using the SYBR Premix Ex Taq Kit (Takara, Tokyo, Japan) and TaqMan microRNA assays (Applied Biosystems, Foster City, CA, USA). The 2^-ΔΔCt^ method was selected to calculate the relative expression level of target genes. The primers are shown in Supplementary Table 2.

### Western blot analysis

Proteins were extracted from cell and tissue lysates according to the manufacturer's instructions. Western blotting was performed as previously described [30].

### MTT assays

Cells were seeded into 96-well plates (1 × 10^4^/well). After administration of 5-HT, SB204741 or Yap-siRNA transfection, MTT (Sigma-Aldrich, St. Louis, MO, USA) was added to the platelets for staining at 37°C for 4 h, and 150 μl of DMSO (Sigma) was subsequently added after discarding the culture medium. The absorption value was measured at OD490.

### Cell cycle and cell apoptosis assays

To study the effects of the 5-HT-Yap pathway on the cell cycle, cells were seeded in 6-well plates with treatments. Next, cells were collected and fixed in 70% ethanol at −20°C overnight. Subsequently, the cells were incubated with PI staining solution (50 μg/mL, Sigma) containing RNase A (100 μg/mL, Sigma) for 20 min at room temperature in the dark. Finally, the cells were analyzed by FACSCalibur (BD Biosciences, Bedford, MA, USA).

For cell apoptosis analysis, the cells treated as presented above were harvested and stained with Annexin V-FITC/PI or Annexin V-PE/7-AAD Apoptosis Detection Kit (KeyGEN BioTECH, Nanjing, China) following the manufacturer's instructions. Stained cells were detected using the FACSCalibur instrument (BD Biosciences).

### Wound healing and cell migration assays

Cells were plated in 6-well plates (5 × 10^5^ cells/well) and then were cultured with 10% FBS to achieve a nearly confluent cell monolayer. A scratch was made carefully by a 10-μl sterile micropipette tip on the cell layer. Next, the cells were washed and the wounded monolayers were photographed to determine the wound width at time 0 h. After administration, the cells were photographed again at 24 h and 48 h.

For the transwell migration assay, the control cells or transfected cells (1 × 10^5^) were added to the top chamber, and the bottom chamber was filled with DMEM containing 5-HT and SB204741 or PD0325901. After fixing, the transwell chambers were stained with 1% crystal violet for 10 min and the cells were counted under a light microscope for quantitation.

### Immunofluorescence assays

To explore the subcellular localization of Yap, immunofluorescence was conducted after treatment with 5-HT, SB204741 or PD0325901. Immunofluorescence was performed as previously described [31].

### Immunohistochemical staining

Immunohistochemical staining was performed using a streptavidin peroxidase-conjugated (SP-IHC) method with Yap or Ki-67 antibodies. Evaluation of the immunohistochemical staining was conducted by two independent pathologists who were blinded to the administration. The assessment criteria were based on a previous study [32].

### *In vivo* experiments

Sixteen female BALB/c nude mice (4–5 weeks old, 14–18 g) were purchased from Animal Feeding Center of Xi’an Jiaotong University Health Science Center. All mice were housed in an IVC system with a controlled environment and temperature (24 ± 1°C). All of the mice were fed standard rodent chow and water ad libitum and were adapted to the environment for 7 days before use. All mice were inoculated subcutaneously by HepG2 cell (1 × 10^5^) suspended in DMEM on the left flank. After subcutaneous injection for 5 days (diameter of xenograft tumor was about 3mm), sixteen nude mice were randomly allocated into 4 groups as follows (4 mice per group): (1) control group: the mice were treated with physiological saline. (2) 5-HT group: the mice were treated with 5-HTP (50 mg/kg) every other day via subcutaneous injection. (3) 5-HT+SB204 group: the mice were treated with 5-HTP (50 mg/kg) every other day and SB204741 (10 mg/kg) twice a day via subcutaneous injection, synchronously. (4) 5-HT+PD032 group: the mice were treated with 5-HTP (50 mg/kg) every other day via subcutaneous injection, and PD0325901 (10 mg/kg) once a day via intragastric gavage. Tumor volume was measured twice a week and calculated as (π×length×width×height)/6. All mice were sacrificed after subcutaneous injection for 4 weeks and the xenograft tumors were explanted for weighing and further experiments. Animal care was in compliance with criteria outlined in the Guide for the Care and Use of Laboratory Animals established by the US National Institutes of Health. The study was approved by the Animal Research Committee of Xi’an Jiaotong University Health Science Center.

### Statistical analysis

The data were expressed as means ± SD. The correlation differences between the respective groups were evaluated by either ANOVA or nonparametric test. A *P*-value less than 0.05 was considered to be statistically significant.

## SUPPLEMENTARY MATERIALS TABLES


