# Supplementary material for: Effects and related mechanisms of serotonin on malignant biological behavior of hepatocellular carcinoma via regulation of Yap
Source: Oncotarget. 2017 May 7;8(29):47412–24. doi: 10.18632/oncotarget.17658 (PMC5564575; doi:10.18632/oncotarget.17658)
Supplement: Supplementary file 1 [file oncotarget-08-47412-s001.pdf]

Effects and related mechanisms of serotonin on malignant biological behavior of hepatocellular carcinoma via regulation of Yap

Supplementary Materials

Supplementary Table 1: siRNA

siRNAs	Sequence (5′-3′)
Control siRNA	5′-GCAAGCUGACCCUGAAGUUCAU-3′
Yap-siRNA #1	5′-CCACCAAGCUAGAUAAAGA-3′
Yap-siRNA #2	5′-GAACAUAGAAGGAGAGGAG-3′

Supplementary Table 2: Primers

Target	Sequence (5′-3′)
GAPDH	F : 5′-CGCGAGAAGATGACCCAGAT-3′
	R : 5′-GCACTGTGTTGGCGTACAGG-3′
$5HT_{1A}R$	F : 5′-GGCAATGCCTGCGTGGTTGC-3′
	R : 5′-TGGCGCACAGGTGCAGGATG-3′
$5HT_{1B}R$	F : 5′-CTCCCGCCACCTCCCAGACA-3′
	R : 5′-GGGTGTGCAGCTTCCGGGTC-3′
$5HT_{2A}R$	F : 5′-TTCACCACAGCCGCTTCAA-3′
	R : 5′-ATCCTGTAGTCCAAAGACTGGGATT-3′
$5HT_{2B}R$	F : 5′-GGCTGATTTGCTGGTTGGATTG-3′
	R : 5′-GGGCCATGTAGCCTCAAACATG-3′
$5\text{-}HT_4R$	F : 5′-GTGCTAAGGTATACAGTTTTGC-3′
	R : 5′-CCAGGGACTCTGGGTCATTG-3′
$5\text{-}HT_5R$	F : 5′-GCCGCTGTGTGTGGTGCTCT-3′
	R : 5′-GACGGTGGCGTGACGGACAG-3′
$5\text{-}HT_7R$	F : 5′-ATTGTGTCCCTGGCGCTGGC-3′
	R : 5′-CGAGGCCGTGCAGCACATGA-3′
E-cadherin	F : 5′-TAACCGATCAGAATGAC-3′
	R : 5′-TTTGTCAGGGAGCTCAGGAT-3′
Vimentin	F : 5′-GAGAACTTTGCCGTTGAAGC-3′
	R : 5′-GCTTCCTGTAGGTGGCAATC-3′
